# Postoperative Peritoneal Granulomatous Inflammation After the Application of Potato Starch-Based Anti-Adhesive Agent in Laparoscopic Endometriosis Surgery

**DOI:** 10.52054/FVVO.15.4.105

**Published:** 2023-12-13

**Authors:** H Krentel, A Naem, A Tannapfel, R Devassy, A.S. Constantin, R.L. De Wilde

**Affiliations:** Department of Obstetrics, Gynecology, Gynecologic Oncology and Senology, Bethesda Hospital Duisburg, Duisburg Germany; Faculty of Mathematics and Computer Science, University of Bremen, Bremen, Germany; Department of Pathology, Ruhr University Bochum, Bochum, Germany; Dr. Rajesh Devassy’s Centre of Excellence in Gynecological Minimal Access Surgery and Oncology, Dubai London Clinic & Specialty Hospital, Dubai, United Arab Emirates; Department of Obstetrics and Gynecology, Albertinen Hospital, Hamburg, Germany; Clinic of Gynecology, Obstetrics and Gynecological Oncology, University Hospital for Gynecology, Pius-Hospital Oldenburg, Medical Campus University of Oldenburg, Oldenburg, Germany

**Keywords:** Adhesions, Adhesiogenesis, Endometriosis, Anti-Adhesive Agents, Inflammation, Granulomatosis

## Abstract

**Background:**

Endometriosis is a chronic inflammatory oestrogen-dependent disease. It is characterised by elevated inflammatory markers in the peritoneal milieu with subsequent adhesiogenesis. Nowadays, excisional, and ablative surgeries are considered the main treatment of endometriosis, and adhesiolysis is being performed almost routinely during these procedures. Postoperative adhesion formation is a significant concern for many surgeons, especially as endometriosis patients are assumed to be predisposed to adhesiogenesis. In order to minimise adhesiogenesis after endometriosis surgery, the usage of different barrier methods have been discussed in the literature. Recent studies aim to investigate the effect of potato starch preparations on adhesion formation in endometriosis patients.

**Objectives:**

We aim to describe the findings of a second-look laparoscopy on patients who received a starch-based anti-adhesive agent.

**Materials and Methods:**

We present a retrospective case series that included the medical, surgical, and histopathologic data of three patients.

**Main Outcome Measures:**

Intraperitoneal adhesion formation and peritoneal inflammation.

**Results:**

All three patients had de-novo adhesions during the second-look laparoscopy. Pathological examination revealed noncaseating granulomatosis of the peritoneum in all patients.

**Conclusion:**

The use of potato starch-based agents as a peritoneal adhesion prophylaxis in laparoscopic endometriosis surgery could lead to granulomatous peritoneal inflammation. Correct application by avoiding powder remnants through complete rinsing and transformation to gel seems to be an important factor to avoid this adverse effect.

**What is new?:**

We aim to highlight that potato starch-based anti-adhesive agents similar to the one used in this study could be a cause of adhesiogenesis and peritoneal inflammation.

## Introduction

Endometriosis is an oestrogen-dependent chronic inflammatory disease characterised by the presence of endometrial-like tissue outside of the uterus. Adhesiogenesis and fibrosis are known to be a substantial element of the endometriosis-related aetiology (Laganà and Naem, 2022; Vigano et al., 2018). Elevated inflammatory markers and increased macrophages II activity were also described in the peritoneal milieu of patients with endometriosis (Halme et al., 1987). Most of these patients undergo at least one laparoscopic surgery in their life. A basic principle in all endometriosis surgeries is the removal of the endometriotic lesions, either by excision or ablation. However, adhesiolysis is also frequently performed in endometriosis patients since most of them present with adhesions involving the pelvic organs (especially the adnexae uteri, sigmoid colon, and rectum), owing to the chronic peritoneal inflammation.

The postoperative adhesiogenesis remains one of the most frequent complications in all types of intraperitoneal interventions (Penzias et al., 2019). Therefore, it is quite plausible to predict that the risk of postoperative adhesiogenesis in endometriosis is even higher because they are already predisposed to adhesions. Adhesions may be symptomatic and also play a major role in subfertility in patients with endometriosis (Tanbo and Fedorcsak, 2017). Different approaches have been developed in order to avoid postoperative adhesion formation in patients with endometriosis, such as transient or permanent ovarian fixation (Dhanawat et al., 2020), consideration of microsurgical principles (Gomel and Koninckx, 2016) and the intraperitoneal application of barrier preparations (Waldron et al., 2022).

Potato starch has been introduced as a potential anti-adhesiogenic agent that can be applied as powder or gel on the surgical field. Recent publications support the use of such potato starch-based preparations to reduce postoperative adhesions (Ziegler and De Wilde, 2022). However, there is also evidence that these preparations may cause inflammation (Ziegler et al., 2021). We present three cases of peritoneal inflammation after the intraperitoneal usage of 4Dry Field®PH (4DF) (PlantTec Medical GmbH, Lüneburg, Germany) in patients with endometriosis and discuss the current literature.

## Materials and Methods

This is a retrospective case series of the postoperative findings of three patients that had excisional endometriosis surgery and received 4DF as an anti-adhesive agent. All three patients underwent laparoscopic complete adhesiolysis and excision of peritoneal endometriosis of the pelvis including deep lesions of the uterosacral ligaments and rectovaginal space. The series did not include patients with ovarian endometriosis or deep endometriosis of the bladder, ureter, or bowel. All three patients were followed up and underwent a second-look laparoscopy to assess the intraperitoneal adhesions after the first operation. Biopsies and subsequent histopathologic examinations of the sites of the newly formed adhesions were performed. Data regarding the patients’ age, symptomatology, their initial surgical management, findings of the second-look surgeries, and the results of the histopathologic examination were collected. A written informed consent was obtained from all three patients regarding the surgical procedures and the inclusion of their medical data in this study. An Institutional Review Board approval was deemed unnecessary to conduct and publish this study since it is based on the retrospective collection of anonymised medical data.

## Results

Three patients underwent laparoscopic surgery for endometriosis due to pelvic pain, dysmenorrhea and infertility. The stage of severity of endometriosis and adhesion was assessed by the revised classification of the American Society of Reproductive Medicine (r-ASRM) and the Enzian classification. We performed complete laparoscopic adhesiolysis and complete excision of endometriotic lesions in all patients. After irrigation, aspiration, and meticulous haemostasis, we used 4DF as a barrier method in order to avoid postsurgical adhesion formation. After covering the surgical site with the powder, we applied ringer lactate to cause gelation.

**Figure 1:**
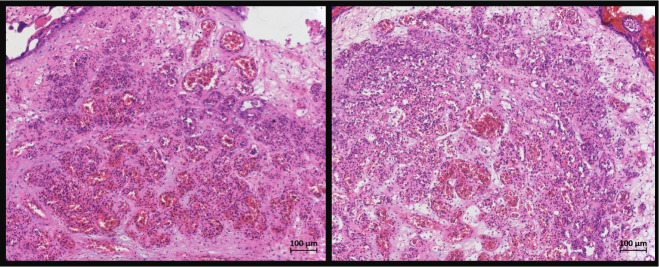
The histopathologic examination of the biopsies taken from the newly formed adhesions. Both images demonstrate noncaseating granulomas that resulted as a reaction against the potato starch powder.

The patients underwent a second look laparoscopy after six to seven months. All patients reported persisting pain despite the administration of hormonal therapy at the time of the second surgery. A summary of the patients’ history and findings is presented in Table I. Upon reoperation, we found peritoneal adhesions and macroscopically visible remnants of the applied powder in combination with a peritoneal inflammation. The histopathological examination of the peritoneal biopsies revealed debris granuloma with macrophages, lymphocytes, and giant cells in all patients (Figure 1).

**Table I:** A summary of the patients’ presentation, symptoms, and time to reoperation.

Case Number	Age (Years)	r-ASRM/Enzian	Symptoms	Procedures during the 1^st^ Surgery	Reoperation Time (Months)
1	36	III A2B2/1	Pelvic Pain and Infertility	Endometriosis Resection. Adhesiolysis. 4DF Application.	7
2	23	II B1/0	Pelvic Pain and Dysmenorrhea		6
3	42	III B1/2	Dysmenorrhea		6

## Discussion

In recent years, several international working groups intended to find a consensus regarding the prevention and reduction of adhesiogenesis after laparoscopic surgery (De Wilde et al., 2017; Diamond et al., 2010). The type of surgery (open or laparoscopic), the type of energy used, the type of the distension medium, the intraperitoneal pressure and the duration of surgery have been identified as factors that can influence postoperative adhesions formation. Most researchers relied on the principles of microsurgery: specifically the importance of minimising iatrogenic injury, ischemia and desiccation of the peritoneum, meticulous haemostasis, excision of necrotic tissue, reduction of cauterisation, prevention of infection and avoiding the introduction of foreign bodies into the abdominal cavity. Although these principles are widely accepted, high quality evidence regarding the efficacy and outcomes of adhesion-preventive techniques is lacking. This is mainly attributed to the limitations and restrictions of research related to adhesions. One reason could be the challenges posed on this research field by national and international regulations (Krielen et al., 2020). Another potential reason could be the lack of a prognostic scoring systems and an internationally accepted adhesions’ classification system for intraperitoneal adhesions. Such classifications would be helpful to identify high risk cases for adhesions formation. There is a persisting demand on the development of safe and effective prophylactic anti-adhesive agents as well as an uncertainty on when to use existing anti- adhesion barriers (Torres-De La Roche et al., 2019).

De Wilde et al. (2022) published an international consensus to establish a base for conducting clinical research on adhesions. The authors highlighted the importance of an appropriate understanding of the mechanisms of adhesiogenesis in order to find an effective prophylactic agent that should be –theoretically- opposing or interfering with the pathophysiology of adhesions (De Wilde et al., 2022).

In endometriosis, adhesions are almost an inevitable part of the disease as they are likely caused by the inflammatory pelvic milieu. However, an important question to be considered is whether these adhesions are comparable to those observed in patients without endometriosis in terms of their causing pathophysiologic mechanisms. It is unknown whether the results of studies conducted on patients without endometriosis could be generalised on patients with endometriosis. Keckstein et al. (2023) recently gave an example on the importance of the surgical technique in preventing adhesion formation. They demonstrated that the use of hybrid argon plasma coagulation reduces the risk of adhesiogenesis compared to sharp excision of endometriosis. Different barrier products are in the market and widely applied to avoid postoperative adhesions (Ahmad et al., 2020). Several case-control studies and one randomised trial showed the benefits of intraperitoneal application of potato starch-based powder and its gel. 4DF is a powder based on purified potato starch that transforms into a gel after application of a solution. The gel then acts as a temporary physical barrier between the surgically traumatised peritoneal surfaces until mesothelial healing is completed. In a rat model, Poehnert et al. (2016) described significant reduction in the incidence and severity of peritoneal adhesion formation after application of 4DF. In the same year, Korell et al. (2016) reported adhesion prevention with the same product in patients with gynaecological surgery. In a second-look laparoscopy of 10 endometriosis patients, the surgeons observed no or only minor adhesions. However, this study did not contain a control group. Moszynski et al. (2022) described the use of potato starch as a haemostatic agent compared to bipolar coagulation in laparoscopic ovarian surgery and showed that the use of modified potato starch might reduce the loss of ovarian reserve due to reduced need of coagulation. Ziegler and De Wilde (2022) also demonstrated a reduced adhesiogenesis after laparoscopic adhesiolysis using 4DF in a retrospective analysis. Krämer et al. (2021) conducted a randomised controlled clinical trial with second-look laparoscopy in 50 endometriosis patients. The authors used 4DF in 25 patients and found a reduction of adhesions by 85% compared to 25 controls. A recent follow-up analysis of the same cohort showed positive results regarding fertility outcomes and pain reduction in the study group (Krämer et al., 2023).

The severity of the disease including all compartments, the coexistence of adenomyosis and the initial presence of endometriosis-related adhesions play a major role in the postoperative outcomes. In addition, the immuno-pathological activity of the peritoneum could differ from one patient to another depending on the endometriotic phenotype and individual genetic patterns. Ziegler et al. (2021) carried out a retrospective comparative analysis of the inflammatory markers in 40 patients undergoing laparoscopic adhesiolysis with and without adhesion prophylaxis with 4DF. The authors reported a significant elevation of postoperative C-Reactive Protein (CRP) levels in patients who received the potato starch-based preparation (Ziegler et al., 2021). In a second- look surgery up to 18 months after the initial intervention, the authors did not report peritoneal inflammation or 4DF remnants in patients without endometriosis. On the contrary, severe peritoneal inflammation with granulomatosis and adhesions were found in the second look laparoscopies of patients with endometriosis who received 4DF prophylaxis during the index surgery. One explanation for these different findings could be the chronic inflammation with persistent repair and remodelling of the peritoneum, as a common feature of endometriosis. Remodelling is achieved through the degradation and reproduction of extracellular matrix components, orchestrated, and guided by extracellular proteolysis and fibroblast activation (Capobianco et al., 2017). The additional application of a foreign body –which is in this case the potato starch-based solution- to the peritoneal cavity might enhance these pathophysiologic effects in patients with endometriosis.

When applying potato starch-based anti- adhesive agents, the surgical site should be covered only by a thin layer of powder and then be rinsed with ringer lactate or saline until the powder becomes a gel and no powder remnants are visible. The exaggerated use of high doses of the powder without sufficient rinsing might be a cause for debris granuloma and consecutive peritoneal inflammation. This is especially important when knowing that starch is classified as a foreign body and as a main cause for granuloma formation (Shah et al., 2017). Therefore, excess starch powder won’t be absorbed by the peritoneum, but induce a chronic inflammation and deteriorate the adhesion status, which may lead to symptoms persistence, inability to appropriately re-operate on patients and other complications.

All recent studies on 4DF have small sample sizes and further larger comparative trials should evaluate the utility of the barrier method in reduction of adhesions and how this correlates to the severity and type of endometriosis. Future trials on adhesion formation should consider the fact that adhesion formation might be different in every disease. Although yet unclear, genetic predisposition to adhesion development apparently exists (Awonuga et al., 2022; Thakur et al., 2021). Although endometriosis is not simply “an ectopic endometrium”, recent studies have demonstrated that it is a disease heavily related to the eutopic endometrium (Laganà and Naem, 2022). The majority of endometrial disorders appear to have genetic and molecular sub-classifications that may influence their management plan (Golia D’Augè et al., 2023). Furthermore, recent views have suggested endometriosis to be a broad term that includes a variety of pathologies that differ in their genetic and epigenetic regulations (Koninckx et al., 2019). Therefore, it would be reasonable to investigate which subtypes of endometriosis are prone to adhesiogenesis, which may guide the application of anti-adhesives to patients with higher tendency for postoperative adhesions formation. This could be a possible future approach by considering the preoperative risk evaluation by using biomarkers or genetic analysis (Guo and Zhang, 2022).

## Conclusions

The use of potato starch-based agents as prophylactics against peritoneal adhesions in laparoscopic endometriosis surgery could induce peritoneal granulomatous inflammation. Correct application by avoiding powder remnants through complete rinsing and complete transformation to gel seems to be an important factor in order to avoid this adverse effect. Larger randomised trials are needed to confirm safety and efficacy of adhesion prevention by potato starch-based preparations in patients undergoing ablative or excisional surgery for endometriosis.
